# Dynamic Sensor Interrogation Using Wavelength-Swept Laser with a Polygon-Scanner-Based Wavelength Filter

**DOI:** 10.3390/s130809669

**Published:** 2013-07-29

**Authors:** Yong Seok Kwon, Myeong Ock Ko, Mi Sun Jung, Ik Gon Park, Namje Kim, Sang-Pil Han, Han-Cheol Ryu, Kyung Hyun Park, Min Yong Jeon

**Affiliations:** 1 Department of Physics, Chungnam National University, Daejeon 305-764, Korea; E-Mails: kyss4133@gmail.com (Y.S.K.); tjdwjdwnd@naver.com (M.O.K.); misun6857@gmail.com (M.S.J.); ikgonss@naver.com (I.G.P.); 2 THz Photonics Creative Research Center, ETRI, Daejeon 305-700, Korea; E-Mails: namjekim@etri.re.kr (N.K.); sphan@etri.re.kr (S.-P.H.); khp@etri.re.kr (K.H.P.); 3 Department of Car-Mechatronics, Sahmyook University, Seoul 139-742, Korea; E-Mail: hcryu@syu.ac.kr

**Keywords:** wavelength-swept laser, fiber Bragg grating, sensor interrogation, strain measurement, semiconductor optical amplifier

## Abstract

We report a high-speed (∼2 kHz) dynamic multiplexed fiber Bragg grating (FBG) sensor interrogation using a wavelength-swept laser (WSL) with a polygon-scanner-based wavelength filter. The scanning frequency of the WSL is 18 kHz, and the 10 dB scanning bandwidth is more than 90 nm around a center wavelength of 1,540 nm. The output from the WSL is coupled into the multiplexed FBG array, which consists of five FBGs. The reflected Bragg wavelengths of the FBGs are 1,532.02 nm, 1,537.84 nm, 1,543.48 nm, 1,547.98 nm, and 1,553.06 nm, respectively. A dynamic periodic strain ranging from 500 Hz to 2 kHz is applied to one of the multiplexed FBGs, which is fixed on the stage of the piezoelectric transducer stack. Good dynamic performance of the FBGs and recording of their fast Fourier transform spectra have been successfully achieved with a measuring speed of 18 kHz. The signal-to-noise ratio and the bandwidth over the whole frequency span are determined to be more than 30 dB and around 10 Hz, respectively. We successfully obtained a real-time measurement of the abrupt change of the periodic strain. The dynamic FBG sensor interrogation system can be read out with a WSL for high-speed and high-sensitivity real-time measurement.

## 1 Introduction

Fiber optic sensors have been of considerable interest in many fields for structural health monitoring of civil infrastructures, buildings, aerospace, and the maritime area. Such sensors mainly use a fiber Bragg grating (FBG) for sensing physical quantities such as strain, temperature, pressure, and vibration in multipoint sensor interrogation systems [1–11]. In optical fiber sensing systems, FBGs have many advantages such as electromagnetic immunity, compactness, remote sensing capability, wavelength selectivity, and easy fabrication. FBGs have been employed as wavelength-selective components capable of selecting wavelengths on an absolute scale [12–16]. The fundamental basis for FBG sensors is interrogation of the shift in the Bragg wavelength of the FBG by using a broadband optical light source. The interrogation of the FBG sensor with a broadband optical source has been implemented with optical filtering techniques that are based on using either an interferometer or a passive optical filter [1–5]. The passive interrogation system using a broadband optical source has a low signal-to-noise ratio (SNR). It is difficult to achieve high-speed dynamic sensing with a broadband optical source. In order to obtain high-speed and high-sensitivity interrogation of a multiple FBG sensor system, the wavelength-swept laser (WSL) has been proposed as a suitable optical source [17]. The WSL has been developed as a promising optical source in optical coherence tomography, optical fiber sensors, and optical beat source generation [7–11,17–27]. The WSL approach has been demonstrated with various methods using a narrowband wavelength scanning filter inside a laser cavity, such as a rapidly rotating polygonal mirror, a diffraction grating on a galvo-scanner, and a scanning fiber Fabry-Perot tunable filter (FFP-TF) [19–24]. The main advantage of FBG sensor interrogation with a WSL is that it allows high-speed measurement in the temporal domain. When using a WSL in the FBG sensor interrogation system, there is a linear relationship between the wavelength measurement and the time measurement. The series of reflected wavelengths in the spectral domain exactly correspond to the series of pulse positions of the reflected signals in the temporal domain. Recently, dynamic strain FBG sensor interrogation using a Fourier-domain mode-locked WSL has been reported [8,10]. In these results, the measurement of the dynamic strain was limited to a few hundred Hz. Also, the WSL with a FFP-TF had a nonlinear response in the wave-number domain, since the response of the piezoelectric transducer in the FFP-TF has a nonlinear response to a sinusoidal modulation signal. Therefore, it requires a recalibration process in the wave-number domain [27–30].

In this paper, we propose a high-speed (∼2 kHz) dynamic multiplexed FBG sensor interrogation using a WSL with a polygon-scanner-based wavelength filter around the 1,550-nm band. The output from the WSL is coupled into the multiplexed FBG array. The multiplexed FBG array consists of five FBGs that have different Bragg wavelengths. One of the multiplexed FBGs in the array is fixed on the stage of the piezoelectric transducer (PZT) stack to allow application of the dynamic periodic strain. The periodic reflected signals collected by the photo-detector are digitized using a data acquisition (DAQ) board. The pulse signal from each FBG is acquired using the peak search VI program that is built into LabVIEW^®^ [31]. We successfully obtain a real-time measurement of the abrupt change of the periodic strain. A sinusoidal voltage waveform with an amplitude of 50 V and with a frequency that is varied from 500 Hz to 2 kHz is applied to the PZT stack to assess the dynamic performance. We obtain the fast Fourier transform (FFT) spectra from the sinusoidal waveforms ranging from 500 Hz to 2 kHz.

## 2 Experiments

**Figure 1:**
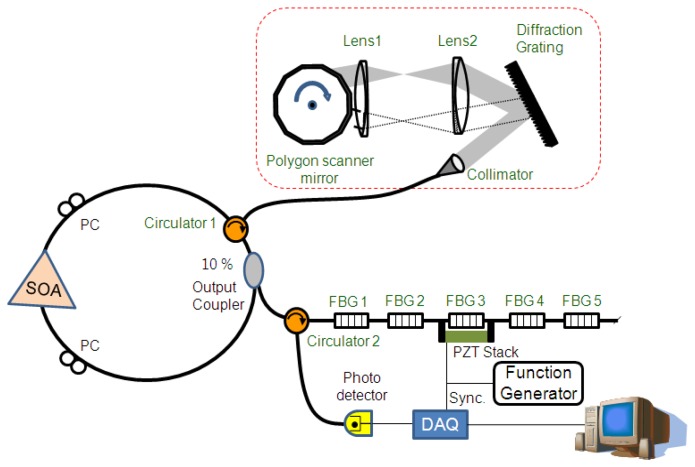
chematic diagram of the experimental setup.

Figure 1 shows the schematic diagram of the experimental setup for a high-speed dynamic sensor interrogation system using a WSL with a polygon-scanner-based wavelength filter. Basically, the WSL consisted of a semiconductor optical amplifier (SOA) as an optical gain medium, two polarization controllers, a 10% output coupler, an optical circulator (labeled as Circulator 1), and a polygon-scanner-based wavelength filter. The center wavelength of the SOA was 1540 nm with a full width at half maximum of 60 nm. The polygon-scanner-based wavelength filter was comprised of a fiber collimator, a blazed diffraction grating with 600 lines/mm at 1,500 nm, two achromatic doublet lenses, and a polygon scanner mirror with 36 facets. The blazed diffraction grating dispersed the collimated beam from the SOA and then recombined the reflected light from the polygon mirror facet [19,20,25]. The output from the WSL was coupled into the multiplexed FBG array through another optical circulator (labeled Circulator 2). The multiplexed FBG array consisted of five FBGs, which had different Bragg wavelengths. The reflected Bragg wavelengths of the multiplexed FBG array were 1,532.02 nm, 1,537.84 nm, 1,543.48 nm, 1,547.98 nm, and 1,553.06 nm. The reflected output from the FBG array was monitored with an optical spectrum analyzer (OSA) via Circulator 2 and with an oscilloscope via a photodiode.

One of the FBGs was fixed on the stage of the PZT stack in order to allow dynamic strain to be applied to it. The reflected Bragg wavelength was shifted when a force is applied to the FBG, changing one of its physical parameter. The reflected signals from the FBGs were acquired via a high-speed photo-detector and a DAQ board (NI5122, National Instruments) that was operated at 100 Msample/s with 14-bit resolution. The trigger signal from a function generator was used to synchronize the DAQ board. The reflected outputs from the five FBGs were simultaneously detected as a series of reflected wavelengths in the spectral domain and as a series of pulses in the temporal domain by scanning over the spectral range. Since there is a correspondence between the time intervals and spectral intervals between the reflected signals from the multiplexed FBGs, the variation of the wavelength of each FBG can be easily converted to account for the sweeping speed of the WSL [7–11,17]. In order to find the period of the variation for the peak points of the reflected signals, the DAQ assistance tool of the LabVIEW^®^ program was used. The pulse signal of each FBG was acquired using the peak search VI of the LabVIEW^®^ program. For tracking of multiple pulse peaks simultaneously, the boundary of the wavelength region should be defined based on the optical bandwidth of the multiplexed FBG array.

**Figure 2:**
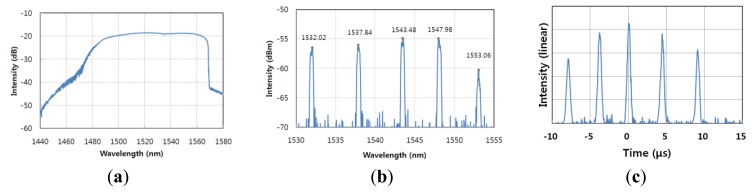
(**a**) Optical spectrum of the WSL; (**b**) optical spectrum of the reflected wavelengths from the multiplexed FBG array; and (**c**) signal of the pulses reflected from the array of multiplexed FBGs.

Figure 2a shows the typical optical spectrum of the output of the WSL. The scanning frequency of the WSL was 18 kHz, and the 10-dB scanning bandwidth was more than 90 nm from 1,475 nm to 1,565 nm at that scanning rate. This covers the full optical bandwidth of the multiplexed FBG array. The instantaneous linewidth of the WSL was about 0.15 nm. This is almost the same as the 3 dB linewidth of the FBGs used. The output power of WSL is more than 0 dBm. Figure 2b shows the optical spectrum of the reflected wavelengths from the multiplexed FBG array. The reflected center wavelengths for FBGs 1–5 were 1,532.02 nm, 1,537.84 nm, 1,543.48 nm, 1,547.98 nm, and 1,553.06 nm, respectively. All of the FBGs had a reflectivity of more than 90%, and the narrow 3-dB bandwidth was measured to be 0.15 nm with the OSA (resolution: 0.1 nm). The reflected outputs from the multiplexed FBG array were converted to a single electrical signal using a high-speed photo-detector. Figure 2c shows the time-domain signal from the reflected pulses from the array of multiplexed FBGs, consisting of five FBGs. Five peaks were observed in the photo-detector output for a single period of the sinusoidal voltage driving the WSL. The positions of the series of pulses from the reflected signals in the temporal domain as shown in Figure 2b exactly correspond to the series of reflected wavelengths in the spectral domain as shown in Figure 2c.

**Figure 3:**
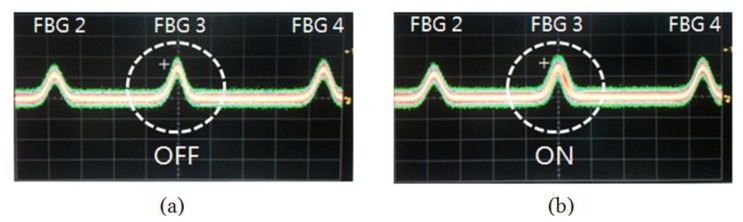
Photograph of the oscilloscope trace (**a**) without dynamic strain and (**b**) with dynamic strain.

**Figure 4:**
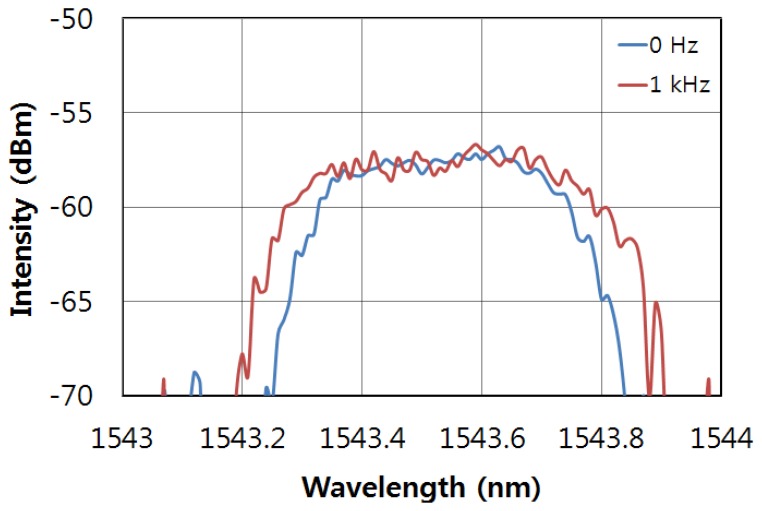
Optical spectrum when dynamic strain (1 kHz) is applied to FBG 3 and in the absence of dynamic strain (0 Hz).

To measure the dynamic strain response, a periodic strain was applied to one of the multiplexed FBGs in the array via the PZT stack. Figure 3a shows a photograph of the oscilloscope trace for the output of the interrogation of the multiplexed FBG array without any dynamic strain. There are three pulses from the reflected signals from the FBGs on the screen of the oscilloscope. When the sinusoidal voltage is applied to the PZT stack, FBG 3 will experience a periodic strain from the PZT stack. A photograph of the oscilloscope trace for the dynamic strain of 1 kHz is shown in Figure 3b. The bandwidth of the center pulse in the photograph of Figure 3b is wider than that of Figure 3a. The pulses of FBG 2 and FBG 4 in the photograph, however, do not show any change for either of the cases of Figure 3a,b. Figure 4 shows the optical spectrum with and without the 1 kHz dynamic strain. The spectral bandwidth of the case with the dynamic strain is wider than that of the case without the dynamic strain. This is due to the modulation from the reflected wavelength of the dynamically strained FBG.

**Figure 5:**
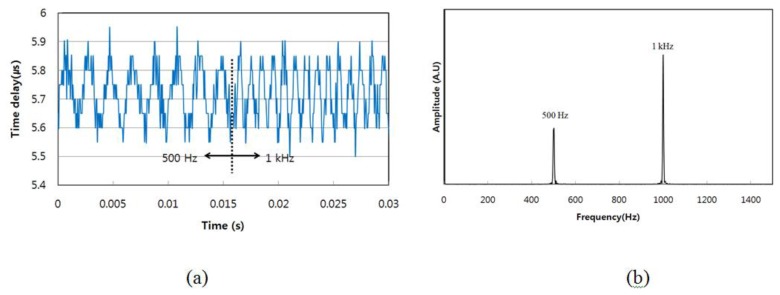
(**a**) Abrupt variation of the periodic strain from 500 Hz to 1 kHz; and (**b**) power spectral density of the FFT spectrum of (a).

In order to confirm the possibility of real-time measurement, the frequency of the sinusoidal waveform applied to the PZT stack was changed abruptly. Figure 5a shows the results of this dynamic measurement when the frequency of the periodic strain in FBG 3 was changed. The periodic reflected signals collected by the photo-detector were digitized using a DAQ board with a high rate of 100 Msamples/s because the sampling rate of the DAQ board determines the accuracy of the acquired data points of the dynamic response. When the frequency of the sinusoidal waveform was changed abruptly from 500 Hz to 1 kHz, the abrupt variation of the periodic strain was captured from the screen of the LabVIEW program, as shown in Figure 5a. It was confirmed that this dynamic sensor system could read out the abrupt change of the periodic strain. The peak-to-peak amplitude of the dynamic applied strain was approximately 285 μɛ. Figure 5b shows the corresponding power spectral density of the FFT spectrum when the frequency of the sinusoidal waveform changed abruptly from 500 Hz to 1 kHz. The FFT spectrum was calculated by using Origin analysis software. The dynamic responses of the peak points at 500 Hz and 1 kHz modulation frequencies are displayed in Figure 5b. The bandwidths for both of them were determined to be around 10 Hz.

**Figure 6:**
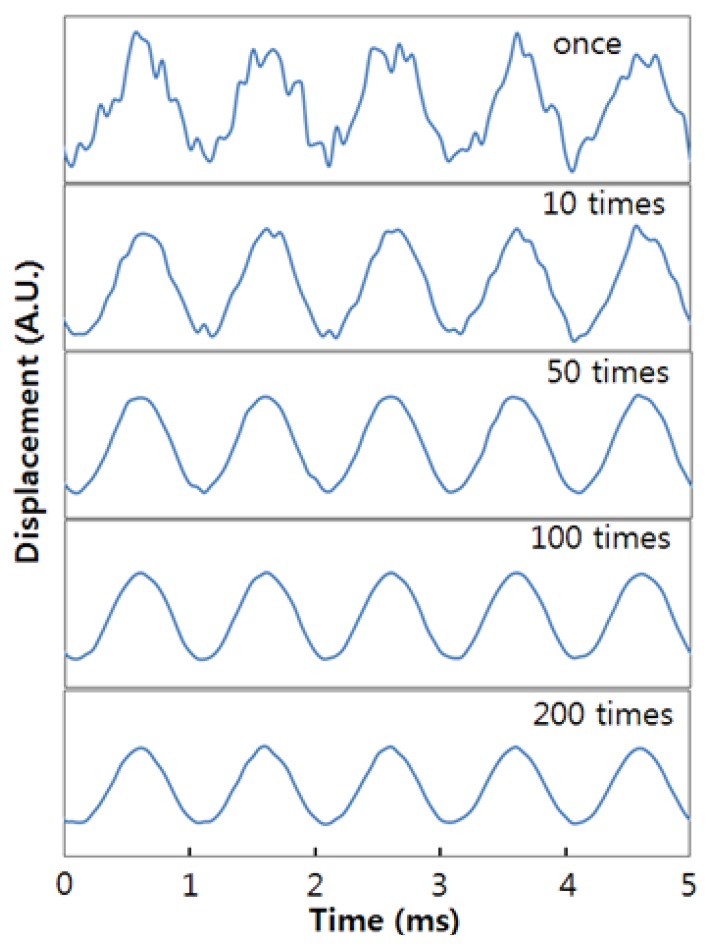
The effect of increasing the number of samples averaged over in measuring the periodic dynamic strain signals.

There is some spectral noise in Figure 5a. This noise is due to the slow response of the dynamic wavelength variation of the FBG. In order to remove the spectral noise, we repeatedly performed the dynamic sensing measurement and then averaged over several tens of samples. The periodic dynamic strain signals were measured for several cases by repeated sampling, as shown in Figure 6. The real-time measurement could be carried out when only one measurement was made. However, successively clearer sinusoidal signals were achieved as the number of measurement samples was increased.

**Figure 7:**
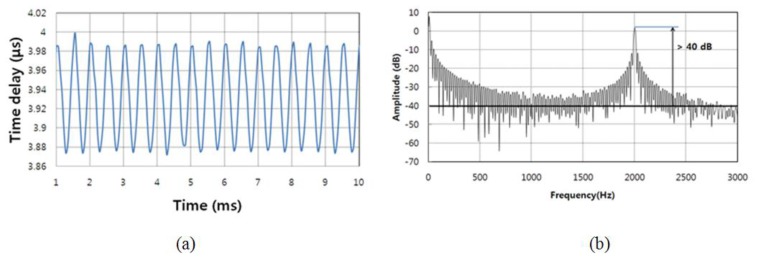
(**a**) Periodic sensor output signal at 2 kHz; and (**b**) power spectral density of the FFT spectrum of (a).

As an example, a sinusoidal waveform with a frequency of 2 kHz and a voltage of 50 V was applied to the PZT stack. By repeatedly measuring the multiple peak positions, the temporal variation of the difference between multiple peaks could be obtained using a LabVIEW peak searching program. The temporal variation of the peak points was repeatedly measured over a total of 100 iterations. The periodic sensor output signal of 2 kHz from FBG 3 in the multiplexed FBG array was achieved using the WSL at a sweeping rate of 18 kHz, as shown in Figure 7a. The DAQ board with the high sampling rate of 100 Msample/s was used to record the temporal variation of the difference between multiple peaks via the photo-detector. In order to improve the resolution, a large number of data points (4,096) are collected for every single sweep of the 18 kHz period. The corresponding FFT spectrum from the periodic output of Figure 7a is shown in Figure 7b. There is a peak for the 2-kHz frequency component in the FFT spectrum. The SNR and frequency bandwidth were determined to be more than 40 dB and around 10 Hz, respectively. The RMS value of the applied strain was calculated as 70.54 μɛ_rms_ at 2 kHz. From the 40-dB SNR at the 2-kHz frequency component, the minimum detectable dynamic strain was calculated as 0.22 μɛ/Hz^1/2^.

**Figure 8:**
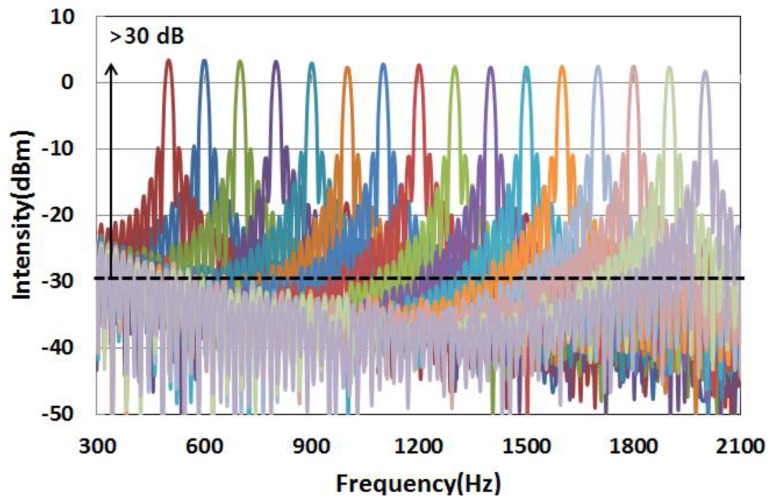
Power spectral density of the FFT spectrum based on varying the frequency of the applied sinusoidal waveform from 500 Hz to 2 kHz.

The sinusoidal waveform with an amplitude of 50 V was applied to the PZT stack with a frequency that was varied from 500 Hz to 2 kHz to assess the dynamic performance of the multiplexed FBG array. The measurement was carried out at intervals of 100 Hz from 500 Hz to 2 kHz. Figure 8 shows the FFT spectra for each of the various applied sinusoidal waveforms, with frequencies from 500 Hz to 2 kHz. The intensity variation of the FFT spectra is less than 2 dB over the whole frequency span. The SNR over the whole frequency span was determined to be more than 30 dB.

## 3 Conclusions

A high-speed (∼2 kHz) dynamic multiplexed FBG sensor interrogation using a WSL with a polygon-scanner-based wavelength filter in the 1,550-nm band has been demonstrated. The scanning frequency of the WSL was 18 kHz, and the 10-dB scanning bandwidth was more than 90 nm at that scanning rate. The output from the WSL was coupled into the multiplexed FBG array, which consisted of five FBGs. A periodic strain was applied to one of the FBGs in the multiplexed array that was fixed on the stage of the PZT stack. A sinusoidal waveform with a frequency that was varied from 500 Hz to 2 kHz was applied to the PZT stack, and the dynamic performance was successfully characterized with a measuring speed of 18 kHz. The SNR and bandwidths over the whole frequency span were determined to be more than 30 dB and around 10 Hz, respectively. We achieved real-time measurement of the abrupt change of the periodic strain without any signal processing delay. Our results confirm that this dynamic FBG sensor interrogation system using WSL can be read out in real time.
